# Preparation and Characterization of an Acid-Responsive ZIF-8 Hydrogel Dressing with Sustained-Release Function for Targeted Therapy of Periodontitis

**DOI:** 10.3390/gels11100813

**Published:** 2025-10-10

**Authors:** Bingbing Chen, Mengqi Hao, Hao Cui, Rui Zeng, Hang Ma, Anying Long, Xuegang Li

**Affiliations:** 1 Engineering Research Center of Coptis Development and Utilization, Ministry of Education, College of Pharmaceutical Sciences, Southwest University, Chongqing 400715, China; double540@email.swu.edu.cn (B.C.); hmq1996@email.swu.edu.cn (M.H.); hangma@swu.edu.cn (H.M.); 2 College of Chemical Engineering, Guizhou University of Engineering Science, Bijie 551700, China; 3 Sichuan Provincial Engineering Research Center of Agricultural and Forestry Waste Resource Utilization, Chengdu Normal University, Chengdu 611130, China; cuihao@cdnu.edu.cn (H.C.); gzsysbk@126.com (R.Z.)

**Keywords:** Hydrogel@ZIF-8@MNZ, drug carrier, pH-responsive, *Fusobacterium nucleatum*, periodontitis

## Abstract

Periodontitis is a chronic oral inflammatory disease whose treatment is often hindered by poor drug retention, prolonged therapeutic regimens, and the rise of antibiotic resistance. In this study, we developed a Hydrogel@ZIF-8@metronidazole (Hydrogel@ZIF-8@MNZ) nanocomposite dressing for targeted, sustained, and in situ antimicrobial therapy. This system integrates ZIF-8, a pH-responsive metal–organic framework, with the antimicrobial agent metronidazole (MNZ), encapsulated within a crosslinked hydrogel matrix to enhance stability and retention in the oral environment. Drug release studies demonstrated that MNZ release was significantly accelerated under acidic conditions (pH 5.0), mimicking the periodontal microenvironment. The Hydrogel@ZIF-8 composite achieved a maximum MNZ adsorption capacity of 132.45 mg·g^−1^, with a spontaneous and exothermic uptake process best described by a pseudo-second-order kinetic model, suggesting chemisorption as the dominant mechanism. The nanoplatform exhibited strong pH-responsive behavior, with enhanced drug release under acidic conditions and potent dose-dependent bactericidal activity against *Fusobacterium nucleatum* (*Fn*). At the highest tested concentration, bacterial survival was reduced to approximately 30%, with extensive membrane disruption observed through live/dead fluorescence microscopy. In summary, the stimuli-responsive Hydrogel@ZIF-8@MNZ nanocomposite offers an intelligent and effective therapeutic strategy for periodontitis. By tailoring its action to the disease microenvironment, this platform enables sustained and localized antibacterial therapy, addressing major challenges in the treatment of chronic oral infections.

## 1. Introduction

*Fusobacterium nucleatum* (*Fn*) is a Gram-negative anaerobic bacterium and a key pathogen implicated in various oral infectious diseases, particularly periodontitis [1,2]. Research has demonstrated that *Fn* contributes to local inflammatory responses by adhering to and invading gingival epithelial cells [3]. Additionally, *Fn* synergizes with other pathogenic species, such as members of the red complex, to significantly accelerate the destruction of periodontal tissues [4]. Under certain conditions, *Fn* can translocate across mucosal barriers to distant tissues and has been associated with several systemic conditions, including atherosclerosis, colorectal cancer, and adverse pregnancy outcomes [5,6,7]. Therefore, effectively controlling the growth and colonization of *Fn* within the periodontal microenvironment is essential for promoting both oral and systemic health.

Despite growing insights into the pathogenic mechanisms of *Fn*, current clinical treatments lack targeted, efficient, and sustained-release antimicrobial strategies for in situ application [8,9]. Traditional approaches, such as mechanical debridement combined with local or systemic antibiotics (e.g., metronidazole, clindamycin), can temporarily suppress microbial populations [10,11]. However, the unique challenges of the oral cavity—such as continuous salivary flow, drug dilution, and biofilm barriers—limit the ability of these agents to maintain effective concentrations, thereby reducing their long-term efficacy. Furthermore, prolonged or broad-spectrum antibiotic use often results in microbial dysbiosis and the emergence of resistant strains, further restricting their clinical utility [12]. Traditional periodontitis treatment primarily involves fundamental procedures such as supragingival scaling and subgingival curettage. However, due to the chronic and recurrent nature of periodontitis, these treatments often require repeated implementation to maintain their efficacy [13]. The extended treatment cycles not only increase the medical burden on patients but also present significant challenges to patient compliance, which may further compromise the overall effectiveness of the therapy. These limitations underscore the urgent need for innovative drug delivery systems capable of providing targeted and sustained antimicrobial action within the oral microenvironment, particularly to improve therapeutic outcomes in chronic infections like periodontitis.

Metal–organic frameworks (MOFs), with their tunable structures, exceptionally high surface areas, and superior drug-loading capacities, have recently emerged as promising candidates for drug delivery and antimicrobial therapy [14,15]. Among these, Zeolitic Imidazolate Framework-8 (ZIF-8)—a zinc-based MOF formed through the self-assembly of zinc ions and 2-methylimidazole—exhibits excellent pH responsiveness and biocompatibility [16,17]. A fundamental requirement for an acid-sensitive carrier is its ability to degrade in acidic environments. However, the high acid resistance characteristic of frameworks such as UIO-66 and MIL-100 makes them unsuitable for this specific function [18,19,20]. In mildly acidic environments, such as those typically found at sites of infection or inflammation, ZIF-8 undergoes gradual degradation, enabling controlled and stimuli-responsive drug release [21]. The oral cavity, with its pH ranging from 5.5 to 7.5 and slightly acidic conditions often observed in periodontal lesions, provides an ideal environment for ZIF-8-based sustained-release systems [22]. Additionally, the cationic surface of ZIF-8 can disrupt bacterial membrane integrity, offering synergistic antimicrobial effects. However, ZIF-8 is susceptible to rapid degradation in the acidic and complex microenvironment typically associated with periodontal lesions, making precise drug release control challenging. Furthermore, due to its relatively fragile crystal structure, the stability and performance of ZIF-8 under prolonged exposure to oral environments, such as saliva and gingival crevicular fluid, have not been sufficiently investigated.

To enhance the stability and retention of ZIF-8 nanoparticles in the dynamic oral environment, recent studies have explored embedding them within three-dimensional hydrogel networks [23]. These hydrogels form adhesive, film-forming layers on gingival tissues or within periodontal pockets, creating in situ drug delivery systems [24]. Known for their excellent biocompatibility and ability to perform in moist conditions, hydrogels provide a stable platform for localized and sustained drug release while minimizing premature drug removal by saliva [25,26]. Inspired by microstructural design principles and bacterial chemotaxis, the concept of a “self-propelled nanorobot” has been proposed [27]. This system integrates responsive ZIF-8 nanoparticles within a hydrogel matrix, enabling autonomous degradation, penetration, and directional drug release within acidic lesion environments. Such a design facilitates active and targeted antimicrobial therapy without the need for external energy input. This innovative strategy opens new avenues for the development of intelligent, targeted, and long-acting therapeutics for periodontitis, offering significant potential to overcome the limitations of traditional treatments.

In this study, we developed a Hydrogel@ZIF-8@metronidazole (Hydrogel@ZIF-8@MNZ) nanocomposite designed to provide targeted, sustained, and in situ antimicrobial therapy against *Fn*. ZIF-8 was utilized as a pH-responsive nanocarrier for metronidazole, enabling controlled drug release under mildly acidic conditions. The ZIF-8 nanoparticles were uniformly embedded within a crosslinked natural polymer hydrogel network, forming a stable and spreadable gel-based delivery platform. The hydrogel’s adhesive and film-forming properties facilitated close contact with gingival tissues and periodontal lesions, significantly improving local drug retention while minimizing the loss of efficacy caused by salivary dilution and mechanical clearance. This study not only presents a novel, stimuli-responsive therapeutic strategy for *Fn*-related periodontal diseases but also lays a theoretical and material foundation for the development of multifunctional nanoplatforms designed for the complex oral environment.

## 2. Results and Discussion

### 2.1. Characterization of Hydrogel@ZIF-8@MNZ

**Figure 1:**
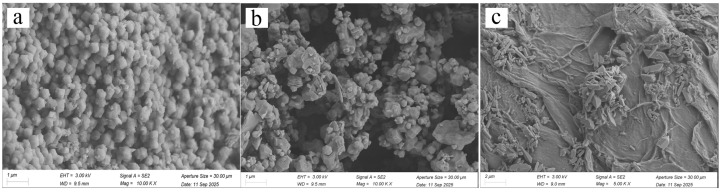
SEM images of materials with different morphologies: (**a**) ZIF-8 crystals, (**b**) ZIF-8 loaded with MNZ, and (**c**) hydrogel-coated ZIF-8 loaded with MNZ.

Figure 1 shows the scanning electron microscopy (SEM) images of materials with different morphologies, including (a) ZIF-8 crystals, (b) ZIF-8 loaded with metronidazole (MNZ), and (c) ZIF-8 loaded with MNZ and encapsulated in hydrogel. Figure 1a displays the cubic morphology of ZIF-8 particles with uniform particle size and smooth surfaces, reflecting the highly ordered crystalline structure of ZIF-8. After MNZ adsorption (Figure 1b), the ZIF-8 particles exhibit noticeable changes. Figure 1b shows increased particle aggregation and rougher surfaces, indicating that the successful adsorption of MNZ has significantly affected the surface properties of ZIF-8. This phenomenon result from the binding of MNZ molecules to the active sites on the ZIF-8 surface through electrostatic interactions or coordination bonds, which alters the surface properties of the material [28]. Similar adsorption behaviors have been reported in other metal–organic framework (MOF) materials, such as the adsorption of Ce by UiO-66 [29]. Figure 1c illustrates ZIF-8 loaded with MNZ and encapsulated in a hydrogel network. The particles are surrounded by the hydrogel, showing a distinct fibrous structure. The introduction of the hydrogel further alters the overall morphology of the material, forming a composite structure. This structure not only enhances the mechanical strength of the material but also provides a protective barrier for sustained drug release. Hydrogel encapsulation is widely used in drug delivery systems, as studies have shown that this method significantly improves the stability and sustained release of drugs [30]. Furthermore, the abundant functional groups in the hydrogel network can further enhance the loading capacity of ZIF-8 for MNZ [31].

Figure S1 presents the FTIR spectra of ZIF-8 and ZIF-8@MNZ. The FTIR spectrum of ZIF-8 exhibits characteristic absorption peaks that confirm the successful formation of its framework structure. Specifically, the peak near 1584 cm^−1^ corresponds to the C=N stretching vibration of the imidazole ring [32], while the multiple peaks observed in the 1350–1500 cm^−1^ range are attributed to C-N and C=C bonds [33]. A strong absorption band in the 900–700 cm^−1^ region is associated with Zn-N coordination vibrations, further supporting the structural integrity of the ZIF-8 framework. In comparison, the FTIR spectrum of drug-loaded ZIF-8@MNZ reveals several new absorption peaks in the 2200–3200 cm^−1^ range (highlighted in yellow), which are characteristic of -N-H, -O-H, and C-H stretching vibrations. Notably, the peaks at approximately 3150 cm^−1^ and 2500–2600 cm^−1^ indicate the successful incorporation of -NH and hydroxyl functional groups from MNZ into the ZIF-8 structure. Additionally, slight shifts and enhanced intensities of peaks within the 1500–1650 cm^−1^ region suggest the presence of hydrogen bonding or other weak interactions between MNZ and the ZIF-8 framework [34].

**Figure 2:**
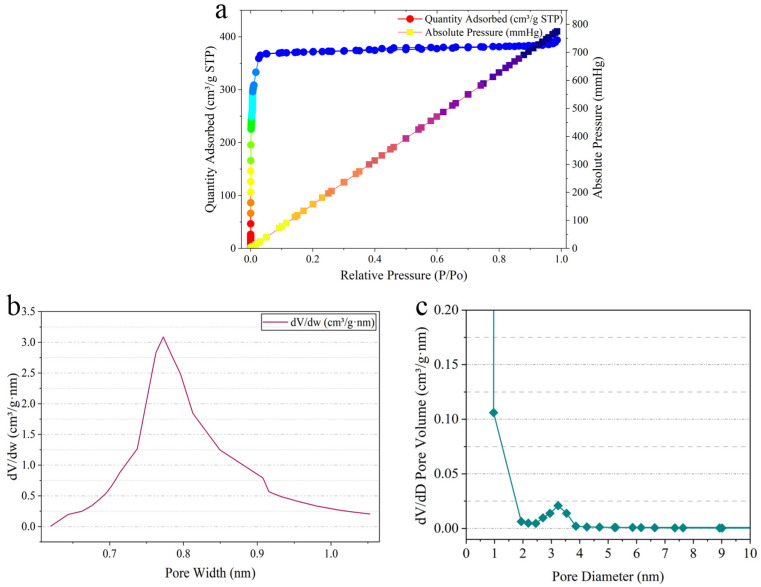
N_2_ adsorption–desorption isotherm of the Hydrogel@ZIF-8 (**a**), along with corresponding pore width (**b**) and pore diameter (**c**) analysis.

As shown in Figure 2a, the N_2_ adsorption–desorption isotherm of Hydrogel@ZIF-8 exhibits a distinct Type I profile, characteristic of a microporous structure. A sharp increase in nitrogen uptake at low relative pressures (P/P_0_ < 0.1) indicates the presence of abundant micropores. The plateau observed at higher relative pressures (P/P_0_ > 0.2) suggests that adsorption is approaching saturation [35]. The minimal or absent hysteresis loop further confirms a narrow pore size distribution and a highly uniform microporous architecture. These observations verify that ZIF-8 encapsulated within the hydrogel maintains its typical sodalite topology, with an estimated pore diameter of approximately 11.6 Å and a fenestration size of 3.4 Å. This isotherm supports the material’s well-defined microporous character. Based on BET analysis, the composite material retains a high specific surface area of 1163.85 m^2^/g and substantial micropore volume, demonstrating excellent MNZ adsorption capacity even after encapsulation within the hydrogel matrix. Figure 2b presents the pore size distribution curve of Hydrogel@ZIF-8, which shows a prominent peak at approximately 0.8 nm, identifying this as the dominant pore diameter. This peak corresponds to the most significant increase in pore volume, indicating that the majority of the material’s pores are centered around this dimension. For pore diameters below 0.8 nm, dV/dw increases with increasing pore size, reflecting the progressive development of micropores. Conversely, for pore diameters above 0.8 nm, dV/dw gradually decreases, suggesting that larger pores contribute less to the overall pore volume. This trend confirms a well-defined microporous structure with a narrow distribution centered around 0.8 nm and a minimal presence of both larger and smaller pores. As shown in Figure 2c, the pore size distribution curve reveals significant contributions to pore volume near 1 nm and in the 2–4 nm range, indicating a bimodal distribution. This structure combines a highly developed microporous network with a moderate presence of mesopores. Such architecture ensures strong adsorption capacity for small organic molecules like MNZ while reducing mass transfer resistance, thereby facilitating efficient diffusion and uptake. Chmelík et al. [36] conducted diffusion studies on methanol in ZIF-8 and found that its porous structure results in a self-diffusion coefficient significantly higher than the macroscopic transport diffusion coefficient. This was attributed to the intrinsic pore architecture and a “cage-hopping” mechanism between adjacent cavities, which reduces convective diffusion resistance and enables faster molecular transport. These features highlight the material’s potential for high-efficiency drug loading and controlled release applications.

SEM and BET analyses revealed that the composite hydrogel films were composed of interconnected nanofibers and bundles, resulting in a hierarchical pore structure comprising micropores, mesopores, and macropores. This multiscale porosity offers dual advantages; the micropores and mesopores originating from ZIF-8 provide a high surface area and efficient drug encapsulation, while the macropores formed by the hydrogel network facilitate nutrient and fluid transport. This hierarchical architecture not only ensures sufficient loading of MNZ but also enables sustained and controlled drug release. Complementary FTIR analysis further confirmed the coexistence of characteristic ZIF-8 vibrations and hydrogel functional groups, validating the successful incorporation of ZIF-8 into the polymeric matrix without compromising its crystalline framework.

**Figure 3:**
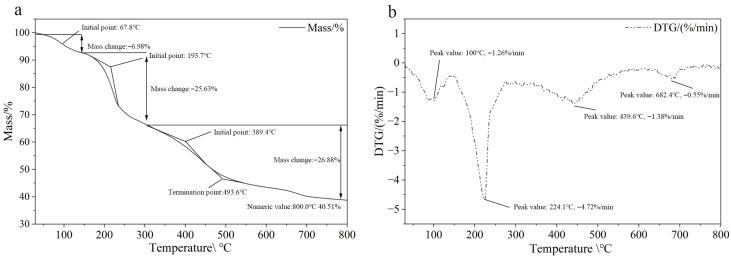
TGA curve (**a**) and DTG curve (**b**) of Hydrogel@ZIF-8, showing its thermal stability and decomposition behavior.

Figure 3a illustrates the TGA and DTG curves of Hydrogel@ZIF-8, highlighting the thermal decomposition behavior and mass loss during heating. The TGA curve reveals three distinct weight-loss stages. The first stage, commencing at approximately 67.8 °C, shows a mass loss of 6.98%, primarily due to the evaporation of physically adsorbed or weakly bound water. The second stage, occurring between 193.7 °C and 389.4 °C, exhibits a mass loss of 25.63%, attributed to the decomposition of hyaluronic acid and partial breakdown of organic ligands. The third stage, beginning at 389.4 °C and concluding around 493.6 °C, involves an additional 26.88% mass loss, corresponding to the structural collapse of the f Hydrogel@ZIF-8 and degradation of residual organic components. Notably, at 800 °C, the sample retains 40.51% of its original mass, reflecting a high inorganic residue content and indicating the thermal stability of the metal oxide components derived from ZIF-8 [37]. This is likely due to the thermal stability of the metal oxide components derived from Hydrogel@ZIF-8. The high residual mass suggests exceptional thermal resistance and highlights the potential for catalytic or adsorption applications at elevated temperatures. Figure 3b depicts the DTG curve of Hydrogel@ZIF-8, which provides insights into the rate of mass loss during thermal decomposition. Distinct peaks in the DTG curve indicate the key temperatures where rapid degradation occurs. A prominent peak at approximately 100 °C (−1.26%/min) corresponds to the rapid evaporation of adsorbed water. The major decomposition event at 241.4 °C (−4.72%/min) is attributed to the breakdown of hyaluronic acid. Additional, smaller peaks at 439.6 °C (−1.38%/min) and 682.4 °C (−0.55%/min) are associated with the collapse of the f Hydrogel@ZIF-8 and a potential high-temperature phase transition, respectively. These findings confirm that the material’s primary decomposition range lies between 200 °C and 500 °C, consistent with its composite structural components. Overall, the DTG analysis emphasizes the high thermal stability of Hydrogel@ZIF-8, solidifying its potential for applications requiring heat resistance and structural integrity.

**Figure 4:**
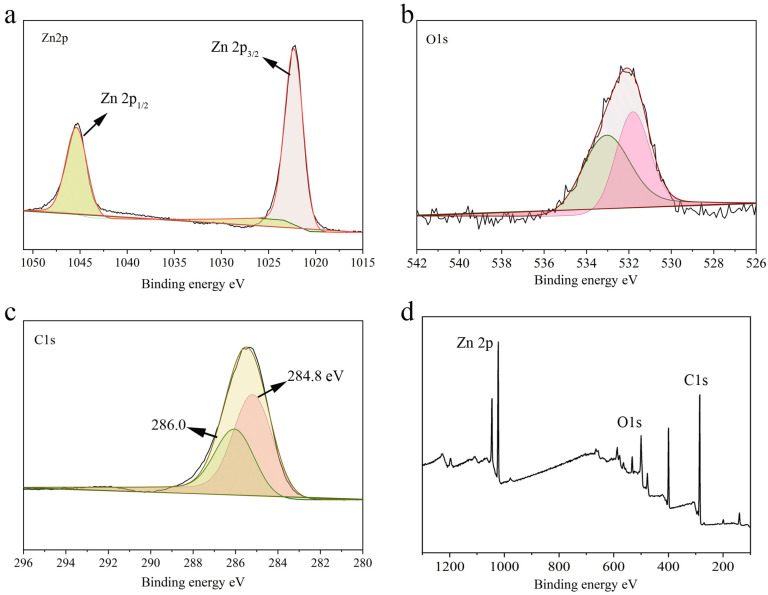
XPS analysis of the as-synthesized Hydrogel@ZIF-8: Zn 2p (**a**), O 1s (**b**), C 1s (**c**), and wide spectra (**d**) core-level spectra.

Figure 4 presents the XPS analysis of Hydrogel@ZIF-8, including full-spectrum scans and high-resolution spectra for Zn 2p, O 1s, and C 1s. The high-resolution Zn 2p spectrum (Figure 4a) exhibits two distinct peaks at approximately 1022 eV (Zn 2p3/2) and 1045 eV (Zn 2p1/2), characteristic of Zn-N coordination [38]. These binding energies confirm that zinc remains primarily coordinated with nitrogen, indicating the preservation of the ZIF-8 framework following its integration into the hydrogel. The high-resolution O 1s spectrum (Figure 4b) reveals multiple peaks in the 530–534 eV range, corresponding to hydroxyl groups (-OH), Zn-O bonds, and adsorbed water. This chemically diverse oxygen environment highlights the hydrophilic nature of the hydrogel, which likely stems from surface -OH groups and moisture adsorption [39]. The high-resolution C 1s spectrum (Figure 4c) shows deconvoluted peaks between 284.8 and 286.0 eV, attributed to C-C/C-H, C-O, and C=O bonds [40,41]. These signals reflect the complex organic composition of the hydrogel matrix, underscoring the presence of various functional groups that may enhance drug loading capacity or facilitate interactions with biological environments. Overall, the XPS results confirm the structural integrity of ZIF-8 within the hydrogel and highlight the functional groups contributing to its hydrophilic and bio-interactive properties.

The full-spectrum XPS scan (Figure 4d) further validates that the primary elements in the Hydrogel@ZIF-8 composite are Zn, O, and C, with no significant impurity peaks, indicating the high purity of the sample. The strong Zn 2p signal confirms the successful incorporation of ZIF-8 into the composite, while the prominent O 1s and C 1s signals highlight the chemical characteristics of the hydrogel matrix. A detailed analysis of the high-resolution spectra for Zn 2p, O 1s, and C 1s indicates that the inorganic ZIF-8 framework remains structurally intact during the composite formation process, while the organic hydrogel matrix is effectively integrated. These findings confirm the successful construction of a multifunctional composite material, combining the thermal stability and framework of ZIF-8 with the hydrophilic and bio-interactive properties of the hydrogel.

Overall, the surface characterization demonstrates that both ZIF-8 and the hydrogel retain their respective chemical functionalities during the assembly process. The preserved Zn-N coordination network in ZIF-8 ensures excellent structural stability, while the hydrogel contributes abundant hydroxyl and carbon–oxygen groups, enhancing its hydrophilic nature. This synergistic combination imparts the composite with remarkable structural integrity, favorable hydrophilicity, a porous architecture, and a strong drug-loading capacity, making it a promising material for various biomedical and catalytic applications.

**Figure 5:**
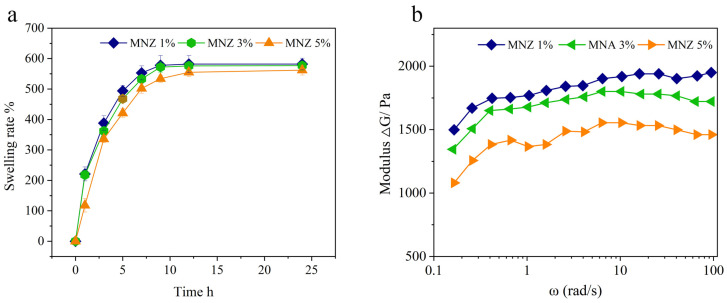
Swelling behavior (**a**) and rheological properties (**b**) of Hydrogel@ZIF-8@MNZ at different drug loadings (1%, 3%, and 5%).

Figure 5 illustrates the swelling behavior and rheological properties of Hydrogel@ZIF-8@MNZ composites with varying ZIF-8 loadings (1%, 3%, and 5%). Figure 5a depicts the swelling ratio over time, where all samples exhibit a rapid initial swelling within the first 6 h, followed by a gradual approach to equilibrium. Among the samples, the hydrogel with 1% ZIF-8 loading achieves the highest equilibrium swelling ratio, nearing 600%, while hydrogels with 3% and 5% ZIF-8 loadings display slightly reduced swelling capacities, yet still surpass 500%, indicating excellent water absorption across all compositions. Figure 5b presents the storage modulus (G′) and loss modulus (G″) across a range of angular frequencies, reflecting the viscoelastic solid behavior of the hydrogels. For all samples, the △G (difference between G′ and G″) increases with frequency, signifying enhanced elastic dominance at higher frequencies [40]. Notably, the hydrogel with 1% ZIF-8 loading demonstrates the largest △G gap, indicating a more stable and well-structured gel network. Conversely, the sample with 5% ZIF-8 loading shows the smallest △G difference, suggesting reduced structural integrity and weaker viscoelastic performance at higher ZIF-8 content.

The swelling rate data demonstrate that the incorporation of ZIF-8 significantly influences the hydrogel’s water absorption properties. At lower loading levels (1% and 3%), the hydrogels exhibit relatively high swelling ratios, likely due to the porous nature of ZIF-8, which enhances hydrophilicity and facilitates water uptake. However, at a higher loading concentration (5%), the swelling rate decreases slightly. This reduction can be attributed to excessive ZIF-8 particles occupying the hydrogel matrix, creating a denser network structure that restricts the penetration and diffusion of water molecules [41]. Despite these differences in equilibrium swelling, all hydrogels—regardless of ZIF-8 content—achieve approximately 90% of their maximum swelling within 6 h, suggesting that ZIF-8 affects the extent of swelling but not the swelling kinetics. Rheological data further highlight the impact of ZIF-8 content on the hydrogels’ network stability and viscoelastic behavior. At a 1% ZIF-8 loading, the hydrogel exhibits the highest △G difference, where the storage modulus (G′) exceeds the loss modulus (G″) significantly. This indicates a stable viscoelastic solid dominated by elastic behavior, reflecting a well-organized and resilient network structure. As the ZIF-8 content increases to 3% and 5%, the △G difference diminishes, likely due to particle aggregation disrupting polymer chain interactions and reducing the effective crosslinking density. This leads to a weaker, less elastic network and diminished mechanical integrity. These findings emphasize the balance required in ZIF-8 loading to optimize both water absorption and structural stability.

Therefore, the hydrogel with 1% ZIF-8 loading demonstrated the most favorable viscoelastic properties and network stability, attributed to its well-organized and resilient polymer network. In contrast, higher loadings likely compromised the structural integrity due to particle aggregation and the subsequent disruption of polymer chain interactions, resulting in a denser yet less elastic network. This highlights the importance of optimizing ZIF-8 content to achieve a balance between structural stability and mechanical performance.

### 2.2. Adsorption Performance

#### 2.2.1. Adsorption Isotherm Fitting and Mechanism Analysis

To gain deeper insight into the adsorption behavior of the ZIF-8-loaded hydrogel (Hydrogel@ZIF-8) toward MNZ, four isotherm models—Langmuir, Freundlich, Dubinin–Radushkevich (D-R), and Temkin—were applied. The results from these models allowed for a comparative assessment of their applicability and provided insights into the potential adsorption mechanisms. Key findings are summarized below, with detailed parameters presented in Table 1.

**Figure 6:**
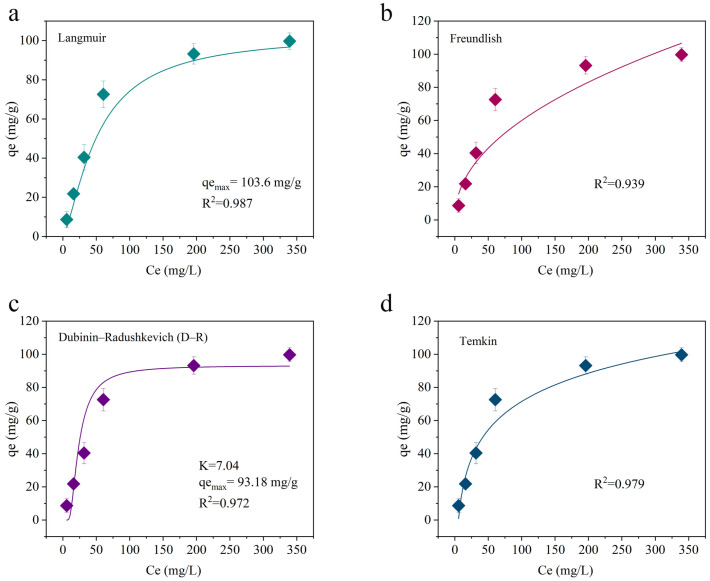
Adsorption isotherm fitting curves of MNZ on Hydrogel@ZIF-8 based on Langmuir (**a**), Freundlich (**b**), Dubinin–Radushkevich (D-R) (**c**), and Temkin models (**d**).

Figure 6 shows the Langmuir isotherm fitting for MNZ adsorption onto Hydrogel@ZIF-8. The Langmuir model assumes monolayer adsorption on a homogeneous surface with no interactions between adsorbed molecules [42]. The fitting yielded a high correlation coefficient (R^2^ = 0.981), indicating that MNZ adsorption may occur in a monolayer manner on uniform active sites of the ZIF-8 surface during certain stages of the process. The maximum adsorption capacity (q_max_) predicted by the model was 132.45 mg·g^−1^, aligning closely with experimental values. Compared to other inorganic carriers, ZIF-8 exhibits an exceptional drug-loading capacity. For example, activated carbon has a maximum drug-loading capacity of 62–102.9 mg·g^−1^, while Mg/Al nanoparticles can load up to 62.8 mg·g^−1^ [43]. Even within the same category of MOF materials, ZIF-8 demonstrates superior performance. The q_max_ of ZIF-8 in this study was 132.45 mg·g^−1^, placing it between that of traditional MOFs (e.g., ZIF-67-SO_4_, 63.03 mg·g^−1^) [44] modified MOFs (e.g., La@ZIF-8, 147.63 mg·g^−1^) [45]. This result indicates that even without additional metal doping or surface functionalization, unmodified ZIF-8 exhibits strong monolayer adsorption potential. In comparison, Fe-ZIF-8 [46] demonstrates a significantly higher q_max_ of 495 mg·g^−1^, highlighting the impact of metal doping and framework modification strategies in substantially enhancing adsorption performance.

The Freundlich model, which describes heterogeneous surfaces and multilayer adsorption, provided the best fit among the four models, with a correlation coefficient of R^2^ = 0.995. This indicates that MNZ adsorption on the Hydrogel@ZIF-8 composite is more complex and occurs on a highly heterogeneous surface. The Freundlich constant (1/*n*) was calculated as 0.417, indicating a favorable and efficient adsorption process (as 0 < 1/*n* < 1). In this study, the Langmuir model provided a better fit to the experimental data than the Freundlich model, suggesting that the adsorption surface was relatively uniform and that monolayer adsorption was the dominant mechanism. While the Freundlich model is widely used in studies involving MOF-like or composite materials—particularly those with heterogeneous surfaces and multilayer adsorption behavior—it was less applicable here. For instance, in the adsorption of heavy metals such as arsenic [47], many studies have shown that the Freundlich model more accurately describes the process due to surface heterogeneity and varying energy sites.

To further investigate the nature of the adsorption forces, the Dubinin–Radushkevich (D-R) model was applied. The calculated mean free energy (E) was 10.72 kJ·mol^−1^, falling within the range of 8–16 kJ·mol^−1^, which suggests a mix of physical adsorption and chemical interactions. Possible chemical interactions include hydrogen bonding, π-π stacking, and surface complexation [48]. The model also showed a good fit (R^2^ = 0.962), confirming the presence of mixed physical and chemical adsorption mechanisms. The Temkin model accounts for adsorbate–adsorbent interactions and assumes that the heat of adsorption decreases linearly with surface coverage. The model yielded a strong correlation (R^2^ = 0.974), with a Temkin constant (b) of 1894.6 J·mol^−1^, suggesting strong interactions (e.g., electrostatic attraction and electron transfer processes) between MNZ molecules and the composite surface. The Temkin model also supports a surface saturation trend, consistent with the experimental observation of rapid initial adsorption followed by a slower approach to equilibrium.

These results confirm that MNZ adsorption onto Hydrogel@ZIF-8 involves a heterogeneous, multilayer process with contributions from both physical and chemical interactions. While the Freundlich model captures the complexity of the composite’s surface characteristics, the other models provide complementary insights into adsorption mechanisms and energetics.

#### 2.2.2. Adsorption Kinetic Model Analysis

**Figure 7:**
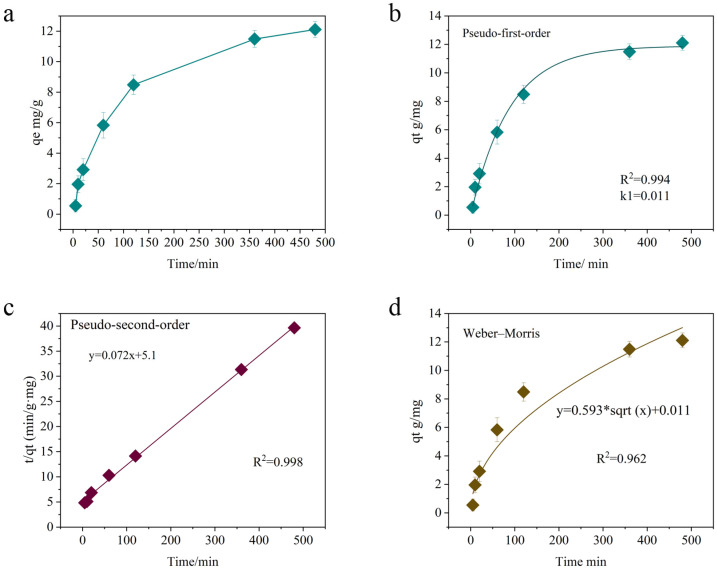
Kinetic analysis of MNZ adsorption on Hydrogel@ZIF-8 (**a**), including experimental adsorption capacity over time and model fits using pseudo-first-order (**b**), pseudo-second-order (**c**), and Weber–Morris (**d**) intraparticle diffusion equations.

To better understand the time-dependent behavior of MNZ adsorption on the Hydrogel@ZIF-8 composite and elucidate the underlying rate-controlling mechanisms, the experimental data were analyzed using three kinetic models: pseudo-first-order, pseudo-second-order, and the Weber–Morris intraparticle diffusion model. The fitting results and corresponding kinetic curves are presented in Figure 7.

The adsorption kinetics of gemcitabine on ZIF-8 were evaluated using three models: pseudo-first-order, pseudo-second-order, and the Weber–Morris intraparticle diffusion model. The pseudo-first-order model, which assumes that the adsorption rate is proportional to the difference between the equilibrium and current adsorption capacities, provided a strong fit to the experimental data (R^2^ = 0.994). However, this model primarily describes physisorption and does not adequately account for the chemisorption mechanisms involved in the process. In contrast, the pseudo-second-order model demonstrated superior accuracy, with an R^2^ value of 0.998. Studies on similar ZIF-8-based materials, such as BSA@ZIF-8 [49] and CCM-ZIF-8 [50], also indicate that the pseudo-second-order model generally provides a better fit than the pseudo-first-order model. This further supports the conclusion that hydrogen bonding and coordination interactions are the key factors governing the adsorption behavior of ZIF-8-based materials.

The Weber–Morris intraparticle diffusion model was applied to evaluate the contribution of internal diffusion to the adsorption process. While the model confirmed the involvement of intraparticle diffusion, it showed a weaker fit to the experimental data (R^2^ = 0.962) compared to the pseudo-second-order model. Studies by Soheila Sharafinia et al. [51] compared the antibiotic adsorption performance of ZIF-8 with other MOF materials and concluded that ZIF-8 undergoes a multi-stage adsorption process, characterized by rapid initial uptake followed by intraparticle diffusion control. Their findings also demonstrated that the pseudo-second-order model provided the best description of adsorption kinetics, while the Weber–Morris model revealed the coexistence of surface adsorption and diffusion limitations. Similarly, Mahdi Hasanzadeh et al. [52] reported that the adsorption of organic pollutants by hydrogel composites followed a multi-phase process. The initial stage was dominated by rapid adsorption at surface-active sites, while the later stage involved slower diffusion of molecules into internal pores or the polymer matrix. The nonlinear fit and intercept in the Weber–Morris model further indicated that external mass transfer resistance in the boundary layer plays a significant role and cannot be ignored [53]. As shown in Figure 7d, this two-step kinetic behavior is consistent with established findings for adsorption in porous materials, where both surface interactions and internal diffusion contribute to the overall process. Additionally, the intercept of the Weber–Morris plot reflects boundary layer effects, indicating the presence of external mass transfer resistance and further supporting the involvement of multiple adsorption mechanisms. This highlights the complex interplay between surface adsorption, diffusion limitations, and mass transfer in the adsorption process.

In conclusion, the pseudo-second-order model is the most suitable for describing the adsorption kinetics of gemcitabine by ZIF-8, as it highlights chemisorption as the primary mechanism. The high R^2^ value and alignment with theoretical expectations reinforce its applicability. While intraparticle diffusion contributes to the process, it is not the dominant factor, as indicated by the Weber–Morris model. These findings align with the adsorption behavior of other pharmaceutical compounds on porous materials, where chemisorption often governs the process. Understanding the dominant mechanism and the contributions of secondary processes provides valuable insights for optimizing the adsorption of gemcitabine in applications such as drug delivery, wastewater treatment, and separation science.

#### 2.2.3. Thermodynamic Analysis

**Figure 8:**
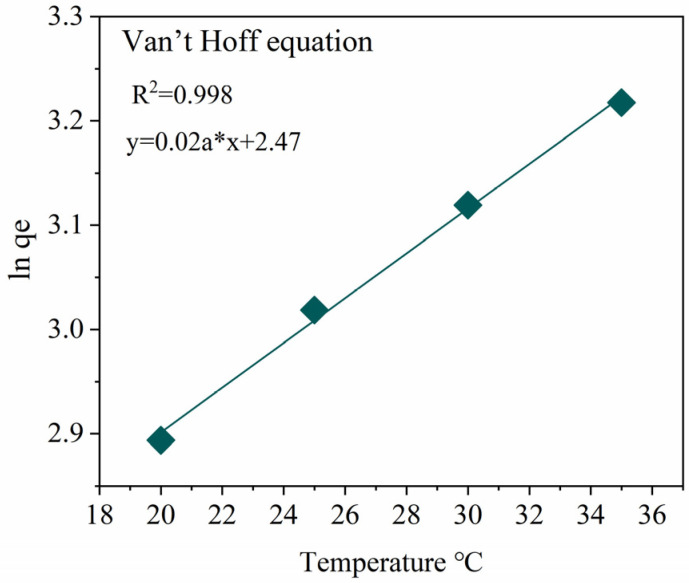
The thermodynamic relationship governing the adsorption of MNZ onto Hydrogel@ZIF-8.

To investigate the effect of temperature on the adsorption of MNZ by Hydrogel@ZIF-8 and to elucidate the thermodynamic characteristics of the process, adsorption experiments were conducted at 298, 308, 318, and 328 K, respectively (Figure 8). The resulting linear Van’t Hoff plot exhibited a negative slope, indicating a negative enthalpy change (ΔH < 0) and confirming that the adsorption process is exothermic. Additionally, the calculated Gibbs free energy change (ΔG) was negative at all tested temperatures, signifying that the adsorption of MNZ onto the Hydrogel@ZIF-8 composite is thermodynamically spontaneous.

The absolute value of ΔG decreased slightly with increasing temperature, indicating a mildly adverse effect of higher temperatures on the adsorption process. This trend aligns with the exothermic nature of the reaction. The calculated enthalpy change (ΔH = −23.74 kJ·mol^−1^) reflects a moderate adsorption heat, which falls within the range typically associated with processes that exhibit characteristics intermediate between physisorption and chemisorption. This observation further supports the findings from the kinetic analysis, suggesting the co-occurrence of both physisorption and chemisorption mechanisms.

A negative entropy change (ΔS = −30.9 J·mol^−1^·K^−1^) was also observed, indicating a reduction in the randomness or disorder of the system during the adsorption process. This decrease in entropy is likely due to the oriented adsorption of MNZ molecules from the solution phase into the confined porous structure of ZIF-8. This transition shifts the system from a state with higher degrees of freedom to a more ordered state, which is characteristic of large molecules binding to surfaces and within the pores of structured materials.

The synthesis of thermodynamic, kinetic, and isotherm model results reveals a comprehensive understanding of the adsorption of MNZ onto Hydrogel@ZIF-8. The adsorption process is spontaneous, as indicated by the negative Gibbs free energy change (ΔG < 0), and exothermic, evidenced by the negative enthalpy change (ΔH < 0). A reduction in entropy (ΔS < 0) suggests an oriented arrangement of MNZ molecules, binding to the material’s surface or within its pore structure and stabilizing the system. The pseudo-second-order kinetic model, with a high R^2^ value of 0.997, highlights chemisorption as the predominant rate-controlling mechanism. Synergistic contributions of both physisorption and chemisorption are further supported by the mean free energy of adsorption (E = 10.72 kJ·mol^−1^) from the Dubinin–Radushkevich isotherm and insights from the Temkin model. The Langmuir isotherm indicates monolayer adsorption nearing saturation, while the Weber–Morris model suggests intraparticle diffusion is not the sole rate-limiting step, with film diffusion also playing a role. In conclusion, the Hydrogel@ZIF-8 composite exhibits excellent adsorption performance for MNZ, governed by a multi-step synergistic mechanism influenced by temperature, porous architecture, and surface chemical properties.

### 2.3. Antibacterial Effect

#### 2.3.1. Sustained Release Rate

**Figure 9:**
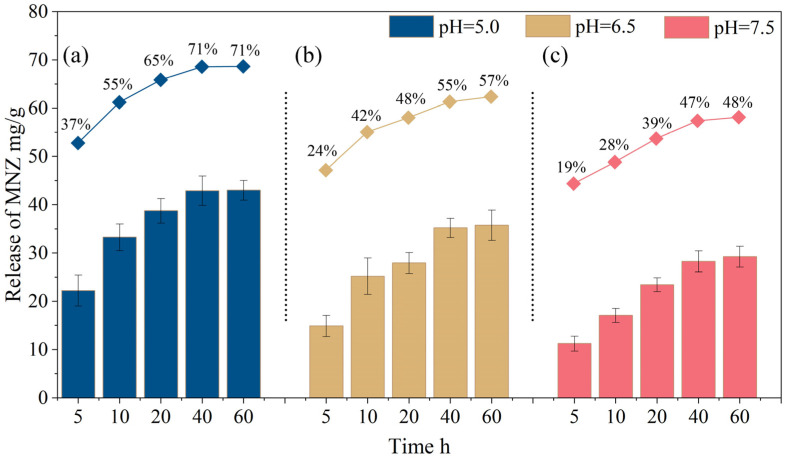
Release profile of MNZ from Hydrogel@ZIF-8 over time at pH values of 5.0 (**a**), 6.5 (**b**), and 7.5 (**c**).

Figure 9 illustrates the release amount and release rate of MNZ (hydrogel-loaded drug) at various time points under pH conditions of 5.0, 6.5, and 7.5. The blue bar graph represents the cumulative release of MNZ (mg/g), while the red line graph depicts the release rate (%) over time. Under pH 5.0 (Figure 9a), MNZ release reached approximately 20 mg/g within the first 5 h and peaked at around 50 mg/g after 60 h, corresponding to a release rate of 70%. Similarly, it has been reported that metronidazole loading onto CF-MWCNTs achieves a maximum release rate of 85% only under extreme pH conditions, such as pH = 1.2. However, the pH in the human oral cavity rarely reaches such extreme levels [54]. At pH 6.5 (Figure 9b), the release was about 15 mg/g within 5 h, eventually reaching 40 mg/g at 60 h, with a release rate of 58%. Under pH 7.5 (Figure 9c), the initial release after 5 h was approximately 10 mg/g, with a final release amount of 35 mg/g at 60 h, corresponding to a release rate of 47%. These findings indicate that the release of MNZ is pH-dependent, with a faster and higher release observed under acidic conditions (pH 5.0) compared to neutral (pH 6.5) or slightly basic (pH 7.5) environments. This behavior suggests that the Hydrogel@ZIF-8 composite could be particularly effective for drug delivery applications targeted at acidic microenvironments, such as those found in inflamed or cancerous tissues.

Figure 9 illustrates the significant influence of pH on the release rate and cumulative release of metronidazole (MNZ) from the Hydrogel@ZIF-8 composite. Under acidic conditions, MNZ showed higher release rates and total release compared to pH 6.5 and pH 7.5 environments. This enhanced release is primarily due to increased hydrogel swelling under acidic conditions, which expands the polymer network and facilitates diffusion. Additionally, the greater ionization and water solubility of MNZ in low-pH environments further promote its release. The results highlight the pH-responsive nature of the Hydrogel@ZIF-8 composite, which could be advantageous for targeted drug release in acidic environments such as inflamed or tumor tissues. To further determine whether the release of MNZ was triggered by the collapse of ZIF-8 or by solution leaching, we conducted XRD analysis on Hydrogel@ZIF-8@MNZ to examine its crystalline structure. As shown in Figure S2, lowering the pH from 7.5 to 5.0 resulted in mixed diffraction peaks within the Hydrogel@ZIF-8@MNZ structure. At pH 6.5, a distinct double peak emerged, marking the onset of structural collapse and mixed-phase formation. Notably, the appearance of a double peak in the low-angle region of MOF diffraction patterns is commonly indicative of structural collapse [55]. At pH 5.0, significant structural changes were observed: peak intensities diminished, reflections disappeared, and the background signal increased, indicating collapse and potential amorphization [56]. These highlighted diffraction regions confirm the framework’s sensitivity to acidic conditions. Given the locally acidic environment in periodontitis lesions [57,58], the pH sensitivity of ZIF-8 enables the Hydrogel@ZIF-8@MNZ structure to collapse in response to decreasing pH, thereby facilitating the targeted release of MNZ at the infection site.

In contrast, at neutral and weakly alkaline pH, the hydrogel matrix remains in a contracted state, reducing its porosity and restricting molecular diffusion. Furthermore, the decreased ionization and solubility of MNZ in these environments result in slower and lower drug release. This pH-responsive behavior is consistent with findings from other MOF-based drug delivery systems. For instance, ZIF-8 nanocarriers have been shown to exhibit accelerated drug release under acidic conditions due to framework decomposition and enhanced drug solubility [59]. Similarly, Jung et al. [60] and Ge et al. [61] demonstrated that ZIF-8/hydrogel composites respond to low pH by swelling, which facilitates drug release. The volumetric expansion of hydrogels induced by pH changes serves as a key mechanism for achieving controlled and targeted drug delivery [22].

The Hydrogel@ZIF-8 composite developed in this study successfully integrates the pH-sensitive swelling properties of the hydrogel matrix with the structural integrity and porosity of ZIF-8. This combination enables rapid and substantial MNZ release under acidic conditions, while effectively minimizing premature release at neutral or alkaline pH. This dual functionality makes the composite highly suitable for targeted drug delivery applications, particularly in environments where acidic conditions, such as inflamed or cancerous tissues, necessitate controlled and efficient release.

#### 2.3.2. Mortality (Minimum Inhibitory Concentration)

**Figure 10:**
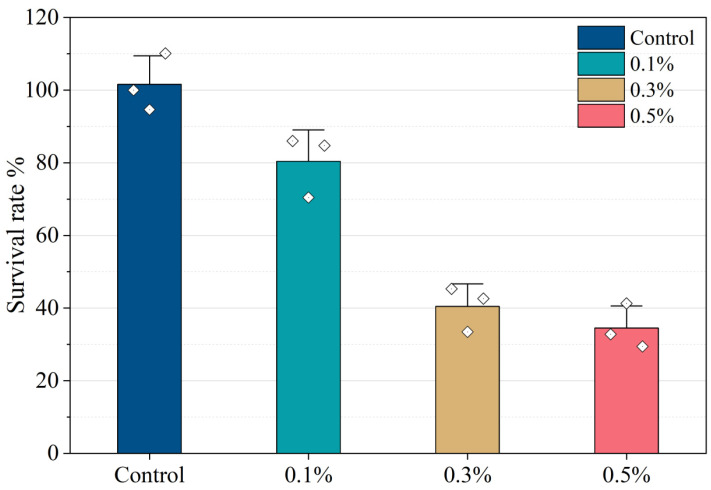
Survival rate of *F. nucleatum* at varying concentrations of ZIF-8@MNZ.

Figure 10 illustrates the effect of varying ZIF-8@MNZ concentrations (0.1%, 0.3%, and 0.5%) on the survival rate of *F. nucleatum*, with the control group normalized to 100% survival. A clear dose-dependent inhibitory trend was observed, where increasing ZIF-8@MNZ concentrations corresponded to a progressive reduction in bacterial survival. Treatment with 0.1% ZIF-8@MNZ resulted in a survival rate of approximately 80%, while 0.3% ZIF-8@MNZ reduced survival to around 50%. The highest concentration tested, 0.5% ZIF-8@MNZ, produced the most pronounced antibacterial effect, reducing the survival rate to approximately 30%. Compared to traditional drug-assisted mechanical debridement, which is limited by rapid drug diffusion and difficulties in maintaining high local drug concentrations [62], the Hydrogel@ZIF-8@MNZ system offers significant advantages. The hydrogel structure improves drug encapsulation efficiency and enables sustained drug release, thereby prolonging therapeutic action while reducing side effects and the frequency of administration. Despite slight variability across replicates, the overall trend confirms the concentration-dependent antibacterial activity of MNZ against *F. nucleatum*.

The inhibitory effect of ZIF-8@MNZ on *F. nucleatum* was clearly concentration-dependent. At 0.1% ZIF-8@MNZ, the reduction in bacterial survival was minimal, indicating that low concentrations exert limited bactericidal activity against this species. However, increasing the ZIF-8@MNZ concentration to 0.3% and 0.5% significantly enhanced its antimicrobial effect, reducing survival rates to approximately 50% and 30%, respectively. This concentration-dependent trend can be attributed to MNZ’s mechanism of action, which involves the generation of reduced metabolites that induce DNA damage in anaerobic bacteria [63,64]. Higher MNZ concentrations produce more active metabolites, thereby amplifying the bactericidal effect [65,66]. These findings align with previous reports on MNZ’s antimicrobial activity against anaerobes. Studies have shown that MNZ exhibits potent bactericidal effects against *F. nucleatum* at concentrations ranging from 1 to 5 μg/mL, with minimum inhibitory concentrations (MICs) typically below 2 μg/mL [67,68]. Additionally, MNZ’s antibacterial activity is enhanced under anaerobic conditions, further reinforcing its selective efficacy against anaerobic pathogens.

#### 2.3.3. Live/Dead Fluorescence Staining to Assess Extracellular Antibacterial Activity

**Figure 11:**
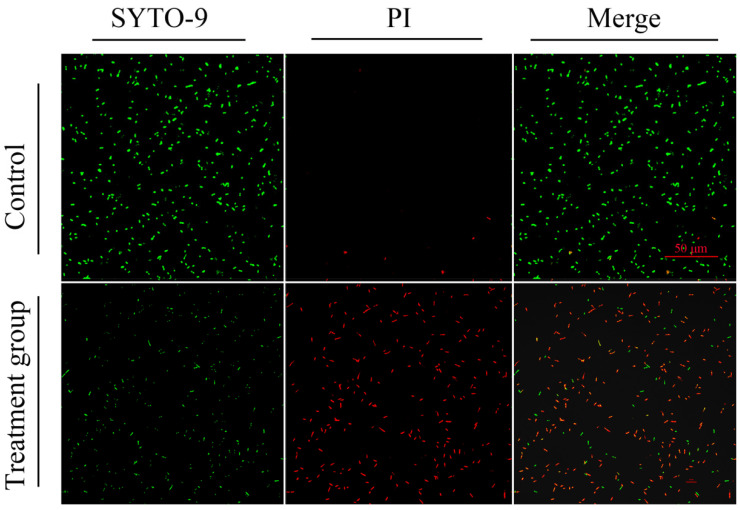
Verification of the extracellular antibacterial effect of MNZ using CLSM.

Figure 11 illustrates the extracellular antibacterial effectiveness of ZIF-8@MNZ through live/dead fluorescence staining, visualized using CLSM. SYTO-9 (green fluorescence) stains live bacteria with intact membranes, while propidium iodide (PI, red fluorescence) penetrates cells with compromised membranes, signifying cell death. The merged fluorescence images combine both channels to provide a clear depiction of the viability status of bacterial cells. In the control group, the merged fluorescence image predominantly exhibited green fluorescence, indicating that most bacteria remained viable, with minimal red fluorescence reflecting limited cell death. In contrast, the ZIF-8@MNZ-treated group displayed a significant increase in red fluorescence, coupled with a notable reduction in green fluorescence. This shift in the fluorescence signal confirms the bactericidal effect of ZIF-8@MNZ, as it significantly increased bacterial mortality. These observations highlight the strong extracellular antibacterial activity of ZIF-8@MNZ against *F. nucleatum*, as evidenced by the marked increase in non-viable cells in the treated group.

SYTO-9 and PI dual-staining analysis clearly demonstrated the bactericidal effect of ZIF-8@MNZ against *F. nucleatum*. In the control group, the predominance of green fluorescence indicated that most bacteria remained viable in the absence of drug treatment. In contrast, the ZIF-8@MNZ-treated group showed a marked increase in red fluorescence, signifying widespread membrane damage and bacterial death. These observations align with MNZ’s well-established mechanism of action, where its reduced metabolites induce DNA damage and disrupt bacterial membrane integrity, ultimately leading to cell death [69]. The fluorescence staining results corroborate the earlier quantitative survival analysis of *F. nucleatum* under varying MNZ concentrations, further confirming the strong antibacterial efficacy of MNZ [70]. These findings are supported by previous studies. For example, Sonja Löfmark et al. [71] demonstrated that MNZ selectively targets anaerobic bacteria via reduction-based metabolic pathways. Similarly, Related research [72,73] has shown that MNZ exhibits potent inhibitory effects on oral pathogens such as *F. nucleatum*, with its bactericidal activity being closely linked to concentration. Compared to traditional antimicrobials, ZIF-8@MNZ offers enhanced specificity and potency, particularly under anaerobic conditions, making it a highly effective agent against anaerobic pathogens.

### 2.4. Antibacterial Effect

pH-Responsive Release Mechanism of ZIF-8 Under Slightly Acidic Conditions: ZIF-8 is a MOF composed of zinc ions coordinated with 2-methylimidazole, forming a robust three-dimensional porous structure. While it remains highly stable in neutral and alkaline environments, ZIF-8 becomes sensitive to slightly acidic conditions [74]. In such environments, the coordination bonds between zinc ions and imidazole ligands gradually break, leading to the degradation of the framework and the subsequent release of the encapsulated drug MNZ. This pH-responsive release behavior is driven by the acid-induced disintegration of the ZIF-8 structure, enabling controlled and targeted drug delivery [75]. Experimental results further confirm that as the environmental pH decreases, the degradation rate of ZIF-8 increases, leading to significantly enhanced MNZ release. This characteristic makes ZIF-8 an ideal carrier for pH-sensitive drug delivery applications, particularly in acidic microenvironments such as inflamed or tumor tissues.

Bactericidal Mechanism of MNZ: MNZ is a broad-spectrum antimicrobial agent that is particularly effective against anaerobic bacteria [71]. Its bactericidal mechanism is closely tied to the anaerobic conditions within these pathogens. Once inside the bacterial cell, MNZ is enzymatically reduced by *Nitroreductase enzymes*, producing reactive metabolites such as nitro radicals. These metabolites interact with bacterial DNA, causing strand breaks and structural damage that inhibit DNA replication and transcription, ultimately resulting in cell death. In addition to DNA disruption, MNZ’s reduced intermediates may also compromise the integrity of the bacterial cell membrane, further enhancing its bactericidal effect. This dual mechanism—DNA damage and membrane disruption—explains the high efficacy of MNZ against anaerobic microorganisms, making it a potent therapeutic option for infections caused by these pathogens.

Synergistic Effect of ZIF-8 Loaded with MNZ: ZIF-8 functions not only as an effective drug carrier but also enhances the performance of MNZ through its unique structural properties. Its highly porous architecture ensures improved dispersibility and stability of MNZ, enabling efficient drug loading and protection. Under slightly acidic conditions, ZIF-8 undergoes controlled decomposition, releasing MNZ in a manner that matches the physiological environments where anaerobic bacteria thrive—such as periodontal pockets and mildly acidic regions of the intestine.

This pH-responsive decomposition allows for targeted drug release precisely at infection sites, maximizing the local concentration of MNZ and enhancing its bactericidal effect. The structural integrity of ZIF-8 in neutral environments, combined with its triggered release in acidic conditions, underscores the synergistic therapeutic advantage of the ZIF-8@MNZ system. Once released, MNZ is taken up by anaerobic bacteria and enzymatically reduced to active metabolites [76], such as nitro radicals. These metabolites cause DNA damage and disrupt bacterial membranes, ultimately leading to cell death. The acidic-triggered release profile of ZIF-8 complements MNZ’s selective anti-anaerobic mechanism, resulting in enhanced drug delivery efficiency and improved bactericidal efficacy [77]. This synergistic effect highlights the potential of the ZIF-8@MNZ system for targeted and effective treatment of anaerobic bacterial infections.

Materials Science Significance: The Hydrogel@ZIF-8@MNZ system offers several significant advantages. First, it features a highly efficient drug release mechanism that is specifically triggered by the acidic microenvironment of periodontal lesions (pH 5.0), while maintaining minimal release under neutral conditions (pH 7.4). Second, the hydrogel matrix encapsulates ZIF-8@MNZ, enabling controlled and sustained drug release. This innovative design addresses the limitations of traditional drug delivery methods, such as rapid diffusion and short duration of action, offering a more efficient and patient-friendly therapeutic solution.

### 2.5. Biological Safety of ZIF-8@MNZ

**Figure 12:**
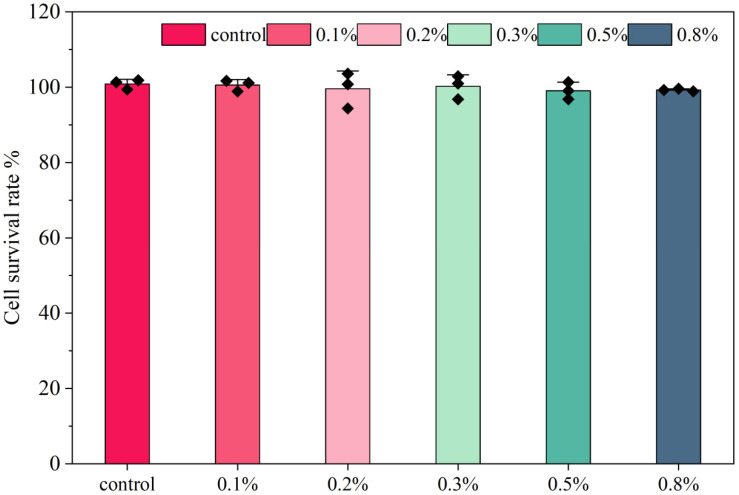
Effect of different concentrations of ZIF-8@MNZ on the viability of RAW 264.7 cells.

After confirming the antibacterial efficacy of ZIF-8@MNZ, we further investigated its cytocompatibility. Evaluating cytocompatibility is a critical step in assessing the biocompatibility of novel materials, as it determines whether the material adversely affects cellular viability and function. As shown in Figure 12, we analyzed the effect of ZIF-8@MNZ on RAW 264.7 cells. The results demonstrated that as the concentration of ZIF-8@MNZ increased from 0.1% to 0.8%, cell viability remained consistently close to 100%. This indicates that within this concentration range, the material exhibits negligible cytotoxicity, showcasing excellent biocompatibility. Additionally, the small error bars in the bar graph reflect high data reproducibility, further validating the reliability of the experimental results. Therefore, it can be concluded that ZIF-8@MNZ is safe within the designed concentration range. Notably, no significant decrease in cell viability was observed with increasing concentration, suggesting that its low toxicity is relatively independent of concentration. Previous studies have shown that metronidazole, as a commonly used antibiotic for anaerobic bacteria, exerts potential cytotoxic effects primarily by inhibiting cell proliferation, interfering with the cell cycle, and inducing oxidative stress-related cellular damage. For instance, research has reported that when metronidazole is conjugated with silver nanoparticles, it induces concentration-dependent cytotoxicity in human osteoblasts and gingival fibroblasts, accompanied by increased ROS production and upregulated expression of necrosis-related marker proteins [78]. Further studies have revealed that the antimicrobial activity of metronidazole can be significantly enhanced when it forms complexes with Cu^2+^ and Zn^2+^ ions [79]. However, its metabolites may induce DNA damage and genetic mutations, particularly in specific bacteria such as Helicobacter pylori. This toxic effect is closely associated with the activity of drug-activating enzymes [80]. In our experiments, no significant cytotoxicity was observed in human-derived cells or murine macrophages. It is noteworthy that Zn^2+^, as an essential trace element for the human body, plays a critical role in various physiological functions. However, excessive exposure to Zn^2+^ may lead to toxic effects. According to guidelines from the U.S. FDA and China’s NMPA, the upper daily intake limit of zinc for adults is 40 mg/day and 45 mg/day, respectively. In this study, the actual Zn^2+^ concentration within the system is far below these thresholds, ensuring that zinc intake does not pose any adverse effects on human health.

### 2.6. Stability of Hydrogel@ZIF-8@MNZ Under Simulated Oral Conditions

**Figure 13:**
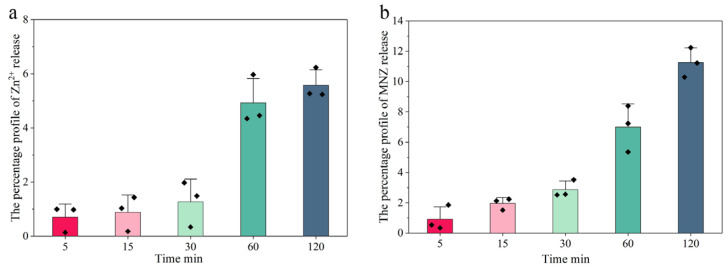
The Zn^2+^ (**a**) and MNZ (**b**) release from Hydrogel@ZIF-8@MNZ over time in simulated healthy oral conditions.

The release behavior of Hydrogel@ZIF-8@MNZ under simulated normal oral conditions (pH 7.0–7.5) demonstrated that ZIF-8 possesses excellent chemical stability, while MNZ exhibited a sustained release profile. As shown in Figure 13a, the cumulative release of Zn^2+^ increased gradually over time, reaching only 1% at 5 min and 7% at 120 min, indicating that the ZIF-8 framework remained largely intact without significant structural collapse. Similarly, Figure 13b shows that MNZ exhibited a slightly faster release rate compared to Zn^2+^, yet its cumulative release remained below 12% within 2 h, demonstrating desirable sustained-release properties.

Throughout the experiment, the stability of ZIF-8 within the Hydrogel@ZIF-8@MNZ system ensured slow carrier degradation. MNZ release exhibited time dependence, with an accelerated release observed particularly between 60 and 120 min, correlating with partial degradation of ZIF-8. Additionally, we investigated the decomposition behavior of ZIF-8 under various pH conditions (Text S1). The results confirmed that ZIF-8 remains stable in neutral environments. Consequently, upon contact with healthy gingival tissue, the structural integrity of ZIF-8 is preserved, suppressing unintended MNZ release and effectively preventing drug wastage.

## 3. Conclusions

This study successfully developed a pH-responsive Hydrogel@ZIF-8@MNZ nano-composite designed for targeted and sustained antimicrobial therapy against *F. nucleatum*, a key pathogen in periodontitis. The composite leverages the pH-sensitive characteristics of ZIF-8 to achieve stimuli-responsive drug release, which is critical for targeting the acidic microenvironment of periodontal infections. Specifically, the system demonstrated a significantly accelerated release of metronidazole (MNZ) at lower pH levels, ensuring drug delivery is concentrated precisely at the site of infection while minimizing off-target effects in neutral or healthy environments. The composite exhibited potent antibacterial efficacy, achieving a substantial reduction in *F. nucleatum* viability in both in vitro and ex vivo models. This is attributed to the synergistic integration of ZIF-8, which provides a stable and protective carrier for MNZ, and the hydrogel matrix, which enhances adhesion to oral tissues and prolongs retention in the oral cavity. Notably, the hydrogel matrix improved the composite’s retention under simulated salivary flow, ensuring sustained drug release and prolonged antimicrobial activity, which are critical for addressing the persistent nature of periodontal infections. By combining the controlled-release properties of ZIF-8 with the bactericidal activity of MNZ, this system offers an intelligent and highly effective therapeutic strategy for managing periodontitis. Beyond its immediate application in periodontal infections, the nano-composite’s microenvironment-specific response makes it a promising platform for other diseases characterized by localized acidic conditions. Future studies should employ multispecies biofilm models, including *Porphyromonas gingivalis*, *Tannerella forsythia*, and *Treponema denticola*, to better replicate the pathological environment of periodontitis and to validate the therapeutic potential of the hydrogel.

## 4. Materials and Methods

### 4.1. Materials

Zinc nitrate hexahydrate (Zn(NO_3_)_2_·6H_2_O), 2-methylimidazole, and metronidazole (MNZ) were purchased from Sigma-Aldrich (St. Louis, MO, USA). Sodium alginate and gelatin (type B) were obtained from Aladdin Reagents (Chengdu, China). All chemicals were of analytical grade and used without further purification. *F. nucleatum* (*Fn*, BNCC337608) was sourced from the BNCC (Beijing, China). The culture medium used was brain heart infusion (BHI) supplemented with 0.5% yeast extract, hemin, and vitamin K1. Tryptic Soy Broth (TSB) was obtained from Sinopharm Chemical Reagent Co., Ltd. (Chengdu, China). SYTO-9 Green Fluorescent Nucleic Acid Stain and DMEM were purchased from Thermo Fisher Scientific (Chengdu, China).

The surface morphology of ZIF-8 crystals, ZIF-8 loaded with MNZ, and hydrogel-coated ZIF-8 loaded with MNZ was examined using scanning electron microscopy (SEM, SU3500, Hitachi, Tokyo, Japan). The crystalline structure was characterized by X-ray diffraction (XRD, Bruker, Karlsruhe, Germany) with Cu Kα1 radiation (λ = 1.5418 Å) over a 2θ range of 5–50°. The functional groups and chemical composition of Hydrogel@ZIF-8@MNZ were analyzed using Fourier-transform infrared spectroscopy (FTIR, Nicolet Summit, Thermo Scientific, Waltham, MA, USA) and X-ray photoelectron spectroscopy (XPS, Thermo Scientific K-Alpha 250Xi, Waltham, MA, USA), respectively. The MNZ content was quantified using high-performance liquid chromatography–mass spectrometry (HPLC-MS, Agilent, Santa Clara, CA, USA), while the Zn^2+^ concentration was determined via inductively coupled plasma mass spectrometry (ICP-MS, PerkinElmer NexION 350D, Waltham, MA, USA). All analyses were performed in triplicate.

### 4.2. Synthesis of ZIF-8 Nanoparticles

ZIF-8 was synthesized using a room-temperature self-assembly method [81]. In brief, 2-methylimidazole (1.6 g) was dissolved in 40 mL of deionized water (solution A), while Zn(NO_3_)_2_·6H_2_O (0.74 g) was dissolved in 20 mL of deionized water (solution B). Solution B was rapidly added to solution A under vigorous stirring. The mixture was stirred continuously at room temperature for 24 h. The resulting ZIF-8 nanoparticles were then collected by centrifugation (10,000 rpm, 10 min), washed three times with ethanol and deionized water, and freeze-dried for storage.

### 4.3. Loading of Metronidazole into ZIF-8

To prepare ZIF-8@MNZ [82], 50 mg of ZIF-8 was dispersed in 10 mL of a metronidazole aqueous solution (10 mg/mL) and stirred for 24 h at room temperature in the dark. The suspension was then centrifuged to collect the drug-loaded nanoparticles, which were washed twice with distilled water to remove any unbound drug and subsequently lyophilized. The drug loading efficiency and entrapment capacity were quantified using HPLC-MS.

### 4.4. Fabrication of Hydrogel@ZIF-8@MNZ

A dual-network hydrogel system was prepared using sodium alginate and gelatin as base polymers [83]. To enhance the suitability of the hydrogel system for drug delivery in the oral cavity, we referred to the study by Simonida Lj. Tomić [84], Sodium alginate (2% *w*/*v*) and gelatin (5% *w*/*v*) were dissolved in deionized water at 50 °C under continuous stirring until a homogeneous solution was obtained. ZIF-8@MNZ nanoparticles (final concentration of 5 mg/mL) were then uniformly dispersed into the hydrogel precursor solution. The composite mixture was poured into molds and cooled to 4 °C to induce physical gelation. Crosslinking was subsequently achieved by immersing the gels in a 100 mM CaCl_2_ solution for 30 min to stabilize the alginate network. The resulting Hydrogel@ZIF-8@MNZ was stored at 4 °C until use.

### 4.5. Adsorption Studies

Adsorption Dynamics: Equilibrium isotherms were obtained by adding 0.1 g of Hydrogel@ZIF-8@MNZ to a series of 50 mL solutions containing 50–1000 mg·L^−1^ metronidazole (MNZ) in a thermostat oscillator set at 37 °C. The solutions were vibrated at 60 rpm for 3 h. Afterward, the Hydrogel@ZIF-8@MNZ was removed, and the concentrations of MNZ in the solutions were determined using HPLC-MS. All experiments were performed in triplicate. To describe the adsorption isotherm characteristics, four commonly used empirical models were applied: the Langmuir isotherm model, the Freundlich isotherm model, the Dubinin–Radushkevich (D-R) isotherm model, and the Temkin isotherm model. These models can be expressed in both nonlinear and linear forms, as shown in Equations (1) and (2):

(1) $$qe=qmax*k1\frac{Ce}{1+k1\times Ce}$$

(2) $$\frac{Ce}{qe}=\frac{1}{qm\times k1}+\frac{Ce}{qm}$$

where qe (mg·g^−1^), q_max_ (mg·g^−1^), qm (mg·g^−1^), k1, and Ce (mg·L^−1^) represent the amount of MNZ adsorbed at equilibrium, maximum adsorption capacity, monolayer adsorption capacity, Langmuir constant, and equilibrium concentration, respectively.

The Freundlich model also has two forms, which may be expressed as

(3) $$qe=k2\times {Ce}^{\left(1/n\right)}$$

(4) $$log(qe)=log(k2)+\frac{1}{n}logCe$$

where qe (mg·g^−1^) is the adsorption amount at equilibrium concentration Ce (mg·L^−1^), and *n* and k2 are the simulation equation constants relevant to the adsorption characteristics.

The Dubinin–Radushkevich (D-R) isotherm model:

(5) $$q_{e}=q_{m}\times e^{\left(-B\times \varepsilon^{2}\right)}\ldots \ldots \ldots \ldots$$

where qe the amount of adsorbate adsorbed per unit mass of adsorbent at equilibrium (mg·g^−1^), qm is the theoretical maximum adsorption capacity (mg·g^−1^), B is a constant related to the mean free energy of adsorption (mol^2^·kJ^−2^), and ε is the Polanyi potential, describing the adsorption energy in porous structures.

The Temkin isotherm assumes that the heat of adsorption of all molecules in the layer decreases linearly with coverage due to adsorbent–adsorbate interactions. The equation is given by

(6) $$q_{e}=B\times \ln\left(A\times C_{e}\right)\ldots \ldots \ldots \ldots$$

(7) $$q_{e}=B\times lnA+B\times lnC_{e}\ldots \ldots \ldots$$

where qe is the amount of adsorbate adsorbed at equilibrium (mg/g), Ce is the equilibrium concentration of adsorbate in solution (mg/L), A is the Temkin isotherm equilibrium binding constant (L/g), and B is a constant related to the heat of adsorption, calculated as

(8) $$B=\frac{RT}{b}\ldots \ldots \ldots \ldots \ldots$$

where R is the universal gas constant (8.314 J/mol·K), T is the absolute temperature (K), and b is the Temkin constant related to the adsorption heat (J/mol)

Adsorption kinetics: The adsorption kinetics were investigated by introducing 0.1 g of Hydrogel@ZIF-8@MNZ into a 50 mL solution containing MNZ at an initial concentration of 100 mg/L. The mixture was subjected to agitation at 60 rpm under a constant temperature of 25 °C for time intervals of 5, 10, 20, 40, and 60 min, respectively. Following the reaction, both the initial and post-reaction solutions were diluted with deionized water, and the concentrations of the target ions were quantified using LC-MS. All experiments were performed in triplicate to ensure reproducibility and reliability.

The experimental results were fitted with pseudo-first-order and pseudo-second-order kinetic models using the following equations.

Pseudo-first-order kinetic model:

(9) $$\ln\left(Qe-Qt\right)=\ln\left(Qe\right)-K_{3}t$$

Pseudo-second-order kinetic model:

(10) $$\frac{t}{Qt}=\frac{t}{Qe}+\frac{1}{K_{4}*{Qe}^{2}}$$

where k3, k4, Qt, and Qe represent the characteristic constant of pseudo-first-order adsorption (min^−1^), characteristic constant of pseudo-first-order adsorption (g·mg^−1^·min^−1^), amount of analyte adsorbed at time t (mg·g^−1^), and adsorption capacity of the adsorbent at equilibrium (mg·g^−1^), respectively.

Weber–Morris intraparticle diffusion model:

qt=kid×t12+C

where qt is the amount of adsorbate adsorbed at time t (mg/g); kid is the intraparticle diffusion rate constant (mg·g^−1^·min^−1/2^); t is the contact time (min); and C is the intercept, which reflects the boundary layer effect (if C = 0, intraparticle diffusion is the sole rate-controlling step; if C > 0, other processes such as film diffusion also influence the adsorption rate). 

#### Integrated Form (Assuming ΔH Is Constant over T)

If ΔH is approximately independent of temperature over the range of interest, integrating the equation gives

$$lnK=-\frac{∆H}{RT}+\frac{∆S}{R}$$

where K = equilibrium constant, T = absolute temperature (in Kelvin, K), ΔH = standard enthalpy changes in the reaction (J/mol), R = universal gas constant (8.314 J⋅mol^−1^⋅K^−1^)

### 4.6. Biological Evaluation Experiments

#### 4.6.1. Swelling Test

The swelling behavior of hydrogels with varying compositions was evaluated following a vacuum freeze-drying pretreatment. A vacuum freeze dryer was employed to ensure complete dehydration and solidification of the hydrogel samples. The initial dry weights of the samples were precisely measured and recorded using an analytical balance. Subsequently, the dried hydrogels were immersed in a simulated saliva solution and incubated at 38 °C within a thermostatic chamber. At predetermined time intervals (1 h, 3 h, 5 h, 7 h, 9 h, 12 h, and 24 h), samples were retrieved, gently blotted with filter paper to remove excess surface moisture, and weighed to determine their swollen weights. The swelling process was continued until the weight of each sample stabilized, signifying that equilibrium swelling had been achieved. Each test was conducted in triplicate, and the results were averaged for statistical reliability. The swelling ratio (SR) was calculated using the following equation:

$$Swelling\;ratio(\%)=\frac{W_{t}-W_{0}}{W_{0}}\times 100\%$$

where W0 is the initial dry weight of the hydrogel and Wt is the swollen weight at time t.

To simulate the varying pH conditions of diseased and healthy tissues, pH values of 5.0, 6.5, and 7.5 were selected to evaluate the release rate of MNZ from Hydrogel@ZIF-8@MNZ under different environments. Additionally, XRD analysis was conducted to examine the crystalline structure, with scans performed over a 2θ range of 5–60°.

#### 4.6.2. Rheological Behavior

The rheological properties of the hydrogels were assessed using a modular intelligent rotational rheometer operated in oscillatory frequency sweep mode [85]. Hydrogel samples with uniform dimensions (thickness: 2 mm; diameter: 8 mm) were carefully prepared using a precision blade and positioned between parallel plates. Rheological measurements were performed across a frequency range of 0.1 to 100 rad/s, maintaining a constant temperature of 37 °C throughout the analysis.

#### 4.6.3. Live/Dead Fluorescence Staining to Assess Extracellular Antibacterial Activity

Bacteria were inoculated into liquid tryptic soy broth (TSB) medium and incubated overnight at 37 °C, with shaking at 80 rpm. Following incubation, the bacterial suspension was washed three times with sterile phosphate-buffered saline (PBS) via centrifugation at 5000 rpm for 5 min. The bacterial concentration was quantified using a UV spectrophotometer. Four experimental groups were established, comprising a blank control and a treatment group. The final bacterial concentration was adjusted to 10^6^ CFU/mL, and the probe concentration was set to 12.5 μg/mL. Samples were irradiated with an 808 nm laser (1.5 W·cm^−2^) for 10 min. After irradiation, the bacterial suspensions were centrifuged at 5000 rpm for 5 min to remove the supernatant. The resulting pellets were resuspended in 200 μL of physiological saline and stained with an equal volume of a mixed dye solution containing PETO-9 (20 μM) and propidium iodide (PI, 100 μM). The samples were incubated at 37 °C for 30 min, washed twice with physiological saline by centrifugation, and finally resuspended in 100 μL of saline. Fluorescence imaging was then conducted using confocal laser scanning microscopy (CLSM).

#### 4.6.4. Cytotoxicity Assay

RAW264.7 cells were seeded into a 96-well plate at a density of 1.0–1.5 × 10^4^ cells per well and cultured overnight to allow stabilization. After removing the old medium, treatment solutions containing 0%, 0.1%, 0.2%, 0.3%, 0.5%, and 0.8% ZIF-8@MNZ (100 μL per well) were added. A carrier control and a positive toxicity control were included. The cells were incubated at 37 °C with 5% CO_2_ for 48 h. Subsequently, 10% of the well volume of CCK-8 reagent was added to each well, and the cells were incubated for an additional 30 min. Absorbance was then measured at 450 nm to evaluate the safety of different concentrations of ZIF-8@MNZ on RAW264.7 cells.

Data analysis was performed using Origin 8.5 and SPSS 21.0 for plotting and correlation analysis. Significant differences between treatments were analyzed using Spearman ANOVA–Duncan.

## Figures and Tables

**Table 1:** Thermodynamic model-related parameters and their significance.

Model	Index	Value	R^2^	Explanation
Langmuir	q_max_ (mg/g)	103.6	0.987	Saturation adsorption capacity; model fit
	KL (L/mg)	0.04		Represents adsorption affinity
Freundlich	KF	3.02	0.939	Multilayer adsorption model; slightly less applicable than the Langmuir model
	*n*	2.3		1 < *n* < 10 indicates favorable adsorption
D-R	q_max_ (mg/g)	93.18	0.972	Slightly lower adsorption capacity
	E (kJ/mol)	6.21		E < 8 kJ/mol indicates a physical adsorption process
Temkin	A (L/g)	1.54	0.979	Adsorption heat varies with surface coverage
	B (J/mol)	214.36		Larger B values indicate higher adsorption heat

## Data Availability

The original contributions presented in this study are included in the article/Supplementary Material. Further inquiries can be directed to the corresponding authors.

## Supplementary Materials

The following supporting information can be downloaded at: https://www.mdpi.com/article/10.3390/gels11100813/s1. Figure S1. FTIR spectrum of ZIF-8 and ZIF-8@MNZ. Figure S2. XRD diffraction patterns of Hydrogel@ZIF-8@MNZ at different pH values: 5.5, 6.5, and 7.5. Text S1. Zn^2+^ release rate during the collapse of ZIF-8. Figure S3. Zn^2+^ release rate during the collapse of ZIF-8 in solution under different pH conditions by ICP-MS.
