# Supplementary material for: Preparation and Characterization of an Acid-Responsive ZIF-8 Hydrogel Dressing with Sustained-Release Function for Targeted Therapy of Periodontitis
Source: Gels. 2025 Oct 10;11(10):813. doi: 10.3390/gels11100813 (PMC12563438; doi:10.3390/gels11100813)
Supplement: Supplementary file 1 [file gels-11-00813-s001.zip › gels-3903010-supplementary.pdf]

Supplemental files

Preparation and Characterization of an Acid-Responsive ZIF-8 Hydrogel Dressing with Sustained-Release Function for Targeted Therapy of Periodontitis

Bingbing Chen [1,2], Mengqi Hao [1], Hao Cui [3], Rui Zeng [3], Ma hang [1], Anying Long [2*], and Xuegang Li [1*]

1 Engineering Research Center of Coptis Development and Utilization, Ministry of Education, College of Pharmaceutical Sciences, Southwest University, Chongqing 400715, China

2 College of Chemical Engineering, Guizhou University of Engineering Science, Bijie, 551700, China

3 Sichuan Provincial Engineering Research Center of Agricultural and Forestry Waste Resource Utilization, Chengdu Normal University, Chengdu 611130, P. R. China

E-mail: xuegangli@swu.edu.cn;longying031042@163.com

Supplemental files Captions

Figure S1. FTIR spectrum of ZIF-8 and ZIF-8@MNZ.

Figure S2. XRD diffraction patterns of Hydrogel@ZIF-8@MNZ at different pH values: 5.5, 6.5, and 7.5.

Text S1. Zn^{2+} release rate during the collapse of ZIF-8

Figure S3. Zn^{2+} release rate during the collapse of ZIF-8 in solution under different pH conditions by ICP-MS.

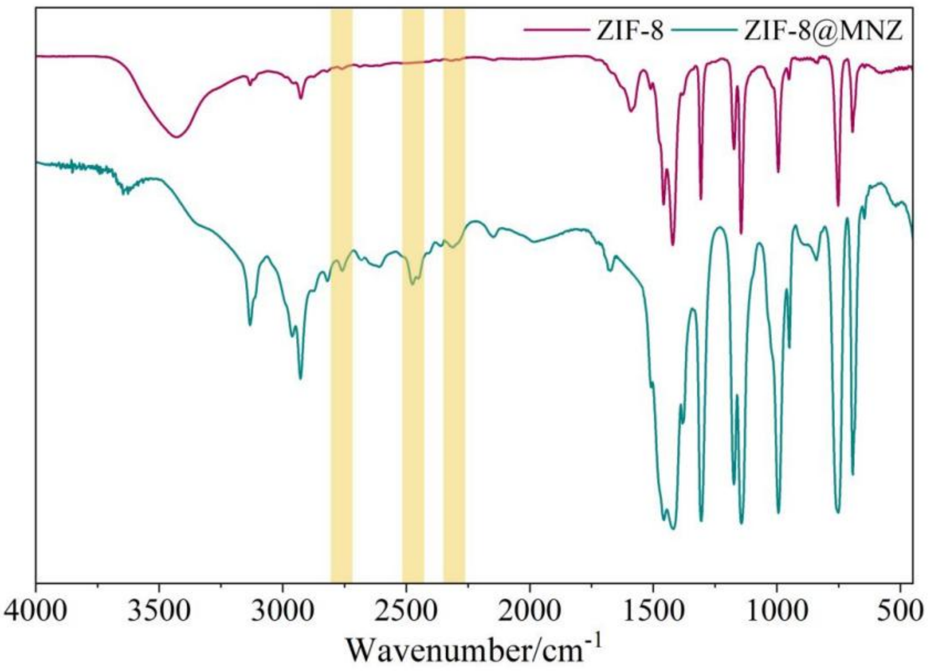

Figure S1. FTIR spectrum of ZIF-8 and ZIF-8@MNZ.

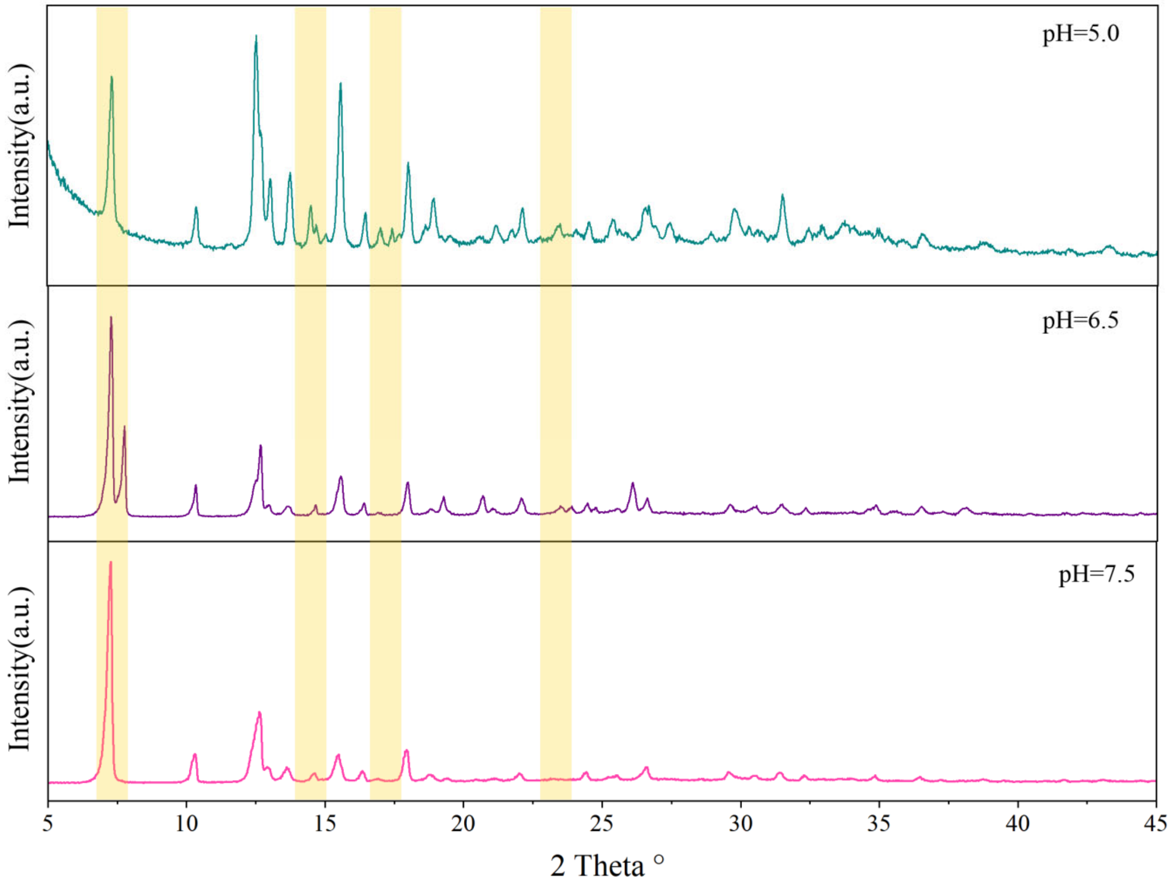

Figure S2. XRD diffraction patterns of Hydrogel@ZIF-8@MNZ at different pH values: 5.5, 6.5, and 7.5.

Text S1. Zn^{2+} release rate during the collapse of ZIF-8

Fig. S3 shows the percentage of Zn^{2+} release during the collapse of ZIF-8 under various pH conditions. The results indicate that under strongly acidic conditions (pH = 1.4 to 4.1), the Zn^{2+} release rate approaches 100%, demonstrating that ZIF-8 is almost completely degraded. Even under mildly acidic conditions (pH = 5.1), the Zn^{2+} release rate remains nearly 100%, suggesting that ZIF-8 is extremely unstable and rapidly releases Zn^{2+} at pH = 5. As the pH increases to 6, the Zn^{2+} release rate significantly decreases to approximately 20%, indicating the improved stability of ZIF-8. At neutral and weakly basic conditions (pH = 7.2 and 8.5), the Zn^{2+} release rate is nearly zero, confirming the excellent stability of ZIF-8 under these conditions. These findings demonstrate that ZIF-8 is highly unstable in acidic environments and can rapidly degrade and release Zn^{2+} even under mildly acidic conditions.

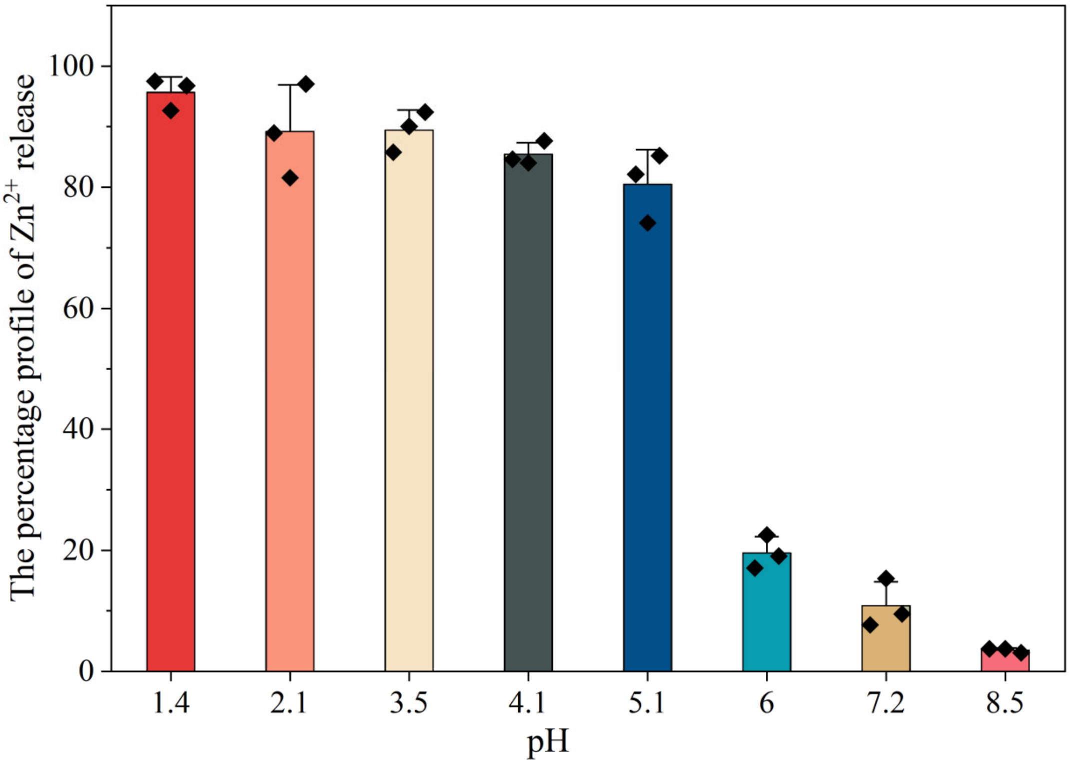

Figure S3. Zn^{2+} release rate during the collapse of ZIF-8 in solution under different pH conditions by ICP-MS.
